# Sensitive and selective colorimetric detection of *Staphylococcus aureus*-*SPA* gene by engineered gold nanosensor

**DOI:** 10.1016/j.sjbs.2023.103559

**Published:** 2023-01-18

**Authors:** Engy madkour, Azza Abou Zeid, Shaimaa Abdel Ghany, Fatimah M. Alshehrei, Doaa EL- Ghareeb, Mohamed Abdel-Hakeem

**Affiliations:** a Department of Pharmaceutical Biotechnology, College of Biotechnology, Misr University for Science and Technology, P. O. Box 77, Giza, Egypt; b Department of Environmental Biotechnology, College of Biotechnology, Misr University for Science and Technology, P. O. Box 77, Giza, Egypt; c Department of Botany and Microbiology, Faculty of Science, Zagazig University, P.O. Box 44519, Sharkia, Egypt; d Department of Biology, Jumum College University, Umm Al-Qura University, P.O Box 7388, Makkah 21955, Saudi Arabia; e Agriculture Genetic Engineering Research Institute (AGERI), Agriculture Research Centre, Egypt

**Keywords:** Gold nanoparticles, *SPA* gene, *Staphylococcus aureus*, Nanosensor, DNA detection

## Abstract

*Staphylococcal* protein A (*SPA*) is an important virulence factor that enables *Staphylococcus aureus* to evade host immune responses. The current work aims to detect the *S. aureus SPA* gene by a colorimetric method based on gold nanoparticles (AuNPs). For this purpose, the chromosomal DNA of *S. aureus* was extracted. Thereafter, primers and thiolated oligonucleotide probe were designed based on protein A sequence data in the gene bank. PCR analysis was performed, and the PCR product was electrophoresed on 2 % agarose gel. Gold nanosensor (Au-Ns) was synthesized by the reaction between AuNPs and the thiolated oligonucleotide probe. The physicochemical properties of AuNPs and Au-Ns were characterized. The detection of the *SPA* gene was performed based on color change detected by the naked eye and UV–vis spectrophotometry. Finally, the described method was optimized and validated for standard, clinical, and food samples. The PCR analysis showed a characteristic fragment of the *SPA* gene with a molecular size of 545 base pairs (bp) and a detection limit of 60 pg/ µL. The physicochemical analyses illustrated Au-Ns’ correct preparation with a zeta potential of −13.42 mV and particle size range 6–11 nm. Moreover, Au-Ns showed 100 % specificity with a detection limit (DL) of 6 fg/ µL. The proposed method was well described to be applied in clinical and research laboratories.

## 1 Introduction

*S. aureus* is a Gram-positive bacterium that is considered one of the most commonly isolated pathogenic bacteria in hospital-acquired infections **(**Mair-Jenkins et al., 2015**)**. It is a major common cause of bloodstream infections, osteomyelitis, endocarditis, and toxic shock syndrome **(**Paulsen et al., 2015**)**. So, the rapid identification of this bacteria is crucial to minimize adverse health impacts. Protein A is the most critical conserved surface protein found in most *S. aureus* strains and plays a significant role in the pathogenesis of this bacteria **(**Amini et al., 2018**)**. The gene that encodes this protein is the *SPA* gene, consisting of 5 conserved domains found at all *S. aureus* strains **(**Kim et al., 2012**)**. Several methods for detecting *S. aureus* have been described. For example, the conventional bacterial culture method can be used for the qualitative and quantitative detection of *S. aureus*. Unfortunately, this technique takes a long time due to the incubation period. The preliminary results often require at least two days, verifying after one week **(**Hameed et al., 2018**)**. Accordingly, rapid techniques have been developed, such as polymerase chain reaction (PCR), enzyme-linked immunosorbent assay (ELISA), and others **(**Liu et al., 2014, Zhu et al., 2021**)**. These advanced methods have helped reduce the detection time to several hours, although there are still several disadvantages, including high cost, complex handling processes, requiring highly specialized laboratories, and lack portability **(**Taravati et al., 2013**)**. Therefore, developing a sensitive and simplified detection method seems imperious to overcome the drawbacks of the current methods.

The colorimetric assay is a method that detects the presence of analytes in a sample through the color change. The results are visible to the naked eye and can be measured quantitatively using a UV–vis spectrophotometer. In the current decade, AuNPs-based colorimetric assay possesses great potential for inexpensive and fast detection of pathogenic bacteria **(**Ahmed et al., 2014**)**. Most reported techniques depend on the basic principle of surface plasmon resonance to detect changes in nanoparticle aggregation states **(**Verma et al., 2015**)**. Colloidal dispersed AuNPs appear red, but their color changes to blue upon aggregation providing a clear colorimetric assay of many analytes **(**Shahi et al., 2021**)**. Recently, researchers have designed various AuNPs-based analytical methods for diagnosing many pathogens such as *Pseudomonas aeruginosa, Escherichia coli, Salmonella enterica*, *S. aureus,* and COVID-19 **(**Agasti et al., 2010, Azzazy et al., 2012, Upadhyayula, 2012, Asif et al., 2020, Alafeef et al., 2021**).** Table 1 summarizes using AuNPs to develop nanosensors to detect bacterial genes. For detecting *S. aureus* colorimetrically, AuNPs have been reported to conjugate Aptamer **(**Zhu et al., 2021, Raji et al., 2021**)**; however, these methods suffer from low-temperature storage conditions and relatively high cost. On the other hand, **(**Houhoula et al., 2017, Shahbazi et al., 2018**)** described utilizing AuNPs-single DNA probe with DL of 123 fg/μL and 8.73 ng/ μL, respectively. The current study aimed to synthesize a novel Au-Ns for detecting *S. aureus* by a colorimetric method using three components: target DNA, Au-Ns, and sodium chloride solution. The synthesized Au-Ns was stable for thirty days. Moreover, the described method was simple, rapid, and provided a unique DL of 6 fg/μL. After optimization and validation, the method was applied to detect the *S. aureus SPA* gene in real clinical and food samples.

**Table1:** Gold nanoparticles-based method for bacterial DNA detection.

Detection method	Microorganisms of interest	Detection limit	Reference
Colorimetric	Salmonella enterica	37 fM	(Kalidasan et al., 2013)
	*Staphylococcus aureus*	500 ng amplicon.	(Chan et al., 2014)
	*Pseudomonas aeruginosa*	9.899 ng/μL	(Amini et al., 2018)
	*Staphylococcus aureus, Listeria monocytogenes, Salmonella Enteritidis*	123 fg/μL	(Houhoula et al., 2017)
	*Staphylococcus aureus*	8.73 ng/μL	(Shahbazi et al., 2018)
	*Shigella* spp.	8.14 ng/μL	(Elahi et al., 2019)
Fluorescence	*Vibrio cholera O1*	2.34 pg/μL	(Narmani et al., 2018)
Electrochemical	*Staphylococcus aureus*	–	(Spain et al., 2011)

## 2 Materials and methods

### 2.1 Materials

*Staphylococcus aureus* (*ATCC 25923*) was used as a standard reference strain throughout the study. *Streptococcus pyogenes, Streptococcus pneumoniae, Kocuria kristinae (Monococcus),* and *Bacillus cereus* isolates were kindly provided by the Microbiology Laboratory, College of Biotechnology Misr University for Science and Technology to be used as negative controls throughout the study. Nutrient Agar, tetrachlorauric acid (HAuCl_4_), and trisodium citrate (Na_3_C_6_H_5_O_7_) were purchased from Sigma-Aldrich. Dithiothreitol (DTT) was purchased from LOBA Chemie.

### 2.2 Bacterial culturing

The bacterial strains were tested for purity by cultivation in Lauria broth (LB) medium at 37 °C for 24 h. After that, growing cultures were streaked on the surface of Baird Parker’s agar for *S. aureus* **(**Corry et al., 2003**)**; 5 % Blood agar for *Streptococcus pyogenes* **(**Shulman et al., 2012**)**, *Streptococcus pneumonia* **(**Mahato et al., 2019**)**, and *Kocuria kristinae* **(**Kandi et al., 2016**)** and Mannitol yolk polymyxin (MYP) agar for *B. cereus* **(**BAM, 1998**)**. Growing colonies on specific media were stained with Gram’s stain and confirmed according to morphological and differential biochemical tests following the key of Bergey’s manual **(**De Vos et al., 2009**)**.

### 2.3 Primer and probe design for *SPA* gene

The primer and probe of the *SPA* gene were designed and verified using the Primer-BLAST tool, NCBI, then synthesized by Willowfort company, United Kingdom **(**Yeh et al., 2012**)**. The obtained primer sequence was Forward: 5′ CGAATCTCAAGCACCGAAAG 3′ Reverse: 5′ CAGGCTTGTTGTTGTCTTCC 3′. The final 20 base thiolated probe sequence was: 5′SH-GCCTAACTTGAACGAAGAAC3′.

### 2.4 DNA extraction

Total DNA of different bacterial species was extracted using Quick DNA™ Miniprep plus kit Cat.no: D4068 (Zymo Research, USA) following the manufacturer’s instructions. Master Mix and DNA marker (Zymo Research, USA).

### 2.5 PCR amplification for *SPA* gene

The presence of the *SPA* gene was determined by PCR assay, starting with the preparation of the PCR mixture with a final volume of 25 μl, contained 1 μl *SPA*-F (10 μM), 1 μl *SPA*-R (10 μM), 2 μl DNA template, 12.5 μl 2 × Taq Master Mix (Zymo Research, USA), and 9.5 μl double distilled water. The PCR conditions were as follow: initial denaturation at 95 °C for 5 min, followed by 35 cycles with a denaturation step adjusted at 94 °C for 30 s. The annealing of the primers to the target DNA sequence was carried at 51 °C for 30 s, and the extension step was at 72 °C for 30 s, while the final extension was 72 °C for 5 min **(**Wichelhaus et al., 2001**).** The electrophoresis analysis of the PCR product was done using 2 % agarose gel. The obtained data were analyzed by Gel pro analyzer software, version 6.

### 2.6 Preparation of AuNPs

Gold nanoparticles were fabricated via the chemical reduction of HAuCl_4_ by sodium citrate **(**Narmani et al., 2018**)**. Typically, 200 ml of 2 mM HAuCl_4_ were heated to boiling for 20 min. To this solution, 10 ml of 1 % (w/v) Na_3_C_6_H_5_O_7_ were gradually added (1 ml/min) with vigorous stirring and heating for 10 min. The obtained red wine solution of gold nanoparticles was stirred for extra 20 min, then cooled down to room temperature and stored at 4 °C for further use.

### 2.7 Synthesis of Au-Ns

The preparation of Au-Ns was carried out according to methods described by Liandris et al., 2009, Shahbazi et al., 2018, with some modifications. Typically, 20 μl of 1 μM of the thiol modified oligonucleotide was initially incubated with 100 μl of DTT (0.1 M) in PBS 100 mM, pH 7.5) at room temperature for 1 h. The excess DTT was removed by washing with 200 μl of ethyl acetate seven times. The activated probe was collected after centrifugation at 12,000xg for 10 min and removing the organic phase. The conjugation step was done by mixing 20 μl activated probe with 1 ml AuNPs followed by incubation at 45 °C for 2 h. The obtained Au-Ns was stored in the dark at 4 °C until use.

### 2.8 Characterization of AuNPs and Au-Ns

The obtained AuNPs and Au-Ns were characterized by size distribution and zeta potential (DLS; Zetasizer Nano ZS, Malvern Instruments, UK), Raman spectroscopy (DXR 2, Thermo Fisher Scientific, Waltham, MA, USA), and Transmission electron microscopy (TEM) (JEOL, JAM-2100-HR-EM).

### 2.9 Colorimetric detection of *SPA* gene

**Fig. 1:**
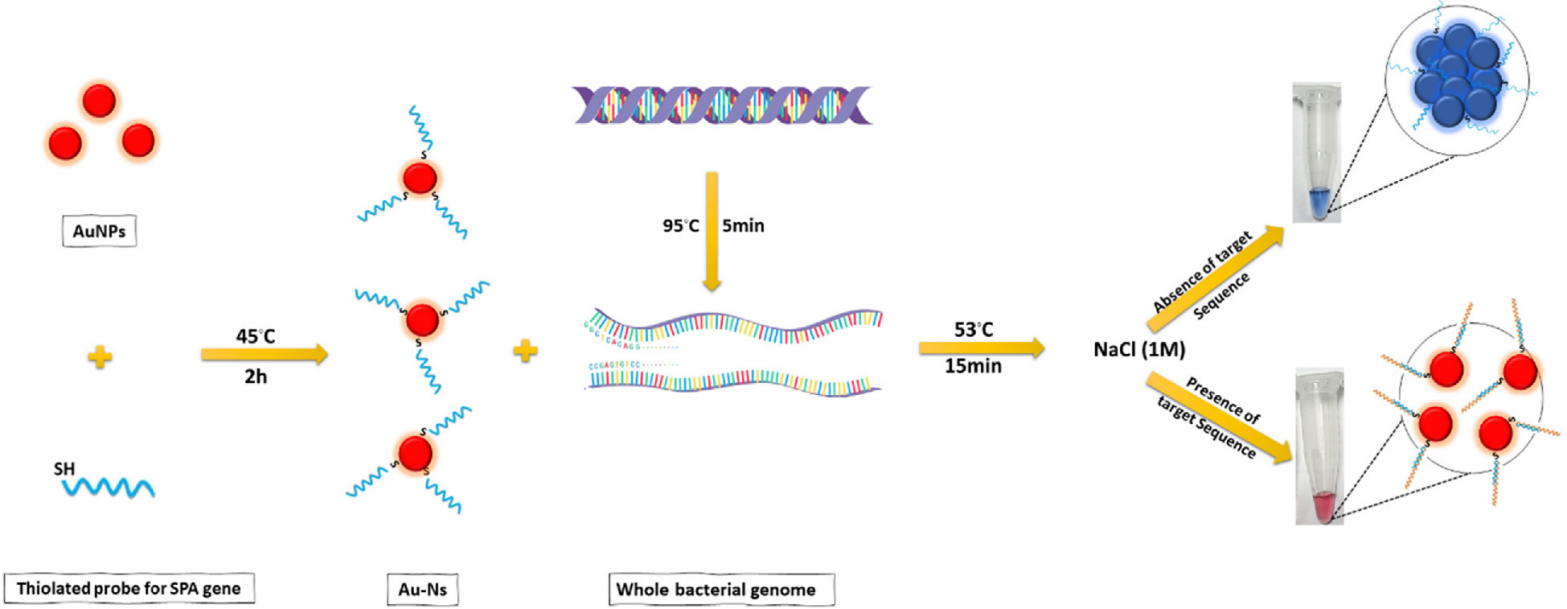
Schematic presentation of the colorimetric detection strategy for detecting *SPA* gene based on Au-Ns.

The preliminary test was done based on the method described by Aldewachi et al., 2017, Shahbazi et al., 2018. Typically, 50 μl of 60 ng/μl *S. aureus* genome was denatured by heating at 95 °C for 5 min, then mixed with 50 μl Au-Ns and incubated for15 minutes. After cooling, 5 μl of NaCl solution was added to the mixture. This step was done in three replicates and characterized by naked eyes and UV–vis spectroscopy (Spectrophotometer, Shimadzu UV1800). For optimizing the method, incubation temperature (23, 35, 45, and 53 °C) and salt concentration (0.1 M – 2 M NaCl) were investigated (Fig. 1).

### 2.10 Methods of validation

The detection limit, specificity, and storage stability were monitored under the optimized assay conditions.

#### 2.10.1 Detection limits

To determine the detection limit (DL) of the Au-Ns for *SPA* gene detection, a range of various concentrations of *S. aureus*-DNA were tested. The extracted DNA was serially diluted with DNase-free water to prepare samples with final concentrations (6 ng/μl, 0.6 ng/μl, 60 pg/μl, 6 pg/μl, 0.6 pg/μl, 60 fg/μl, 6 fg/ μl, and 0.6 fg/ μl). 50 μl from each concentration were tested by the same above method. Blanks containing PBS were compared to the test samples to read the results using a UV–vis spectrophotometer. DL was determined by using equation (1).

(1) $$Detection\;limit=\frac{3.3\;SD}{b}$$

Where SD is the standard deviation of the blank measurements (n = 5) and b is the slope of the calibration curve (Fig. 4c) **(**Amini et al., 2018**)**.

#### 2.10.2 Specificity

*Staphylococcus aureus* ATCC 25923 standard reference strain and the four negative control isolates *(Streptococcus pyogenes, Streptococcus pneumoniae, Kocuria kristinae, Bacillus cereus)* were used for diagnostic specificity of the suggested method. For this purpose, 60 ng/μl of each bacterial DNA sample in addition to Au-Ns blank were tested based on the described method of section 2.9. The obtained color was examined by the naked eye and UV–vis spectrophotometry. The specificity % was calculated by using the following formula:

(2) $$specificity\;\%=\frac{Nt}{Nt+\;Pf}$$

Where Nt is the true negative results (number of negative samples giving blue-colored solution), Pf is false-positive results (number of positive samples, *S. aureus* reference strain, giving blue-colored solution) **(**Zorkafli et al., 2019**).**

#### 2.10.3 Stability

The prepared Au-Ns was stored at 4 °C for 30 days. The storage stability was determined by usage at regular time intervals in the colorimetric detection of the *SPA* gene.

#### 2.10.4 Direct application on clinical and food samples

Isolation of *S. aureus* from different food sources and clinical specimens was performed under aseptic conditions by streaking inocula on Baird Parker agar medium. Then DNA of the obtained *S. aureus* isolates were extracted as described above (*sec*. 2.4) and used to detect the presence of the *SPA* gene.

#### 2.10.5 Isolation of *S. aureus* from clinical specimens

Clinical specimens swabs (ten nasopharynges and ten skin) were kindly obtained from the bacteriology laboratory at Misr University for Science and Technology (MUST) Hospital (Soad Kafafy Specialized Hospital). Swabs were immediately streaked on the surface of Baird Parker agar plates and incubated at 37 °C for 24 h.

#### 2.10.6 *S. aureus* isolation from food samples

Fifty different food samples (15 blue cheese, 5 food additives (Maggi), 15 cheddar cheese, and 15 meat products) were randomly collected from markets in 6th October City – Giza - Egypt. 10 gm of small cut pieces of different food samples were added to 90 ml of sterile saline solution and kept at 60 rpm shaker overnight at room temperature. Loop from each prepared suspension was taken using sterile bacterial metal loops, streaked on the surface of Baird Parker agar plates, and incubated for 37 °C for 24 h.

## 3 Results

### 3.1 PCR amplification of *SPA* gene for *S. aureus* identification

In the current study, PCR analysis was performed to detect the presence of the *SPA* gene and determination of PCR detection limits. The obtained data showed that the first four prepared concentrations (60 ng/μL, 6 ng/μL, 0.6 ng/μL, and 60 pg/μL) of *S. aureus* DNA extracts gave specific bands with the molecular size of 545 bp. So, the detection limit of the PCR-based method was determined as 60 pg/ μL.

### 3.2 Characterization of AuNPs and Au-Ns

**Fig. 2:**
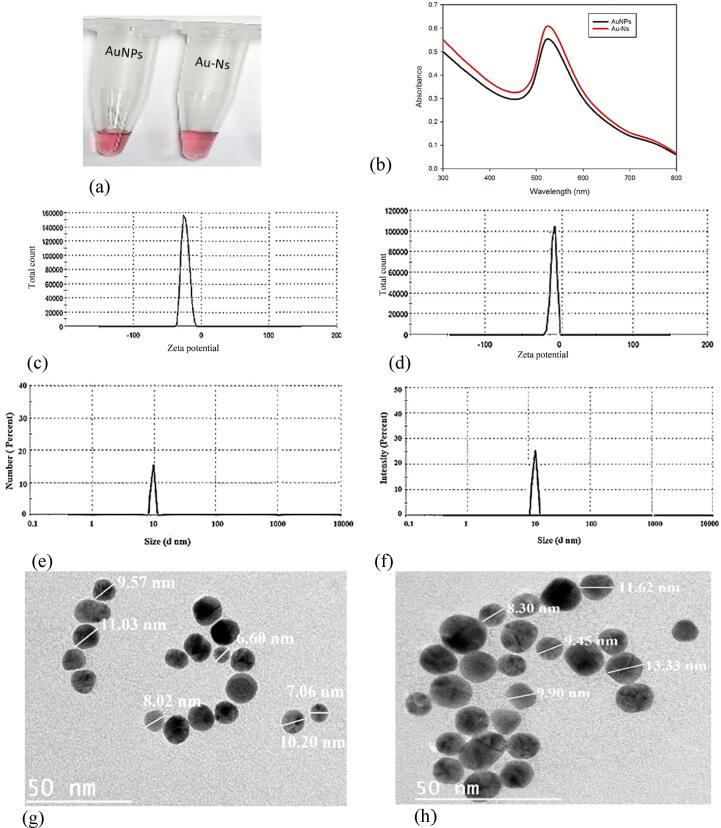
Characterization of nanomaterials. **a.** representative photo of AuNPs and Au-Ns. **b.** UV–vis spectra of the AuNPs and Au-Ns. **c.** zeta potential of AuNPs. **d.** zeta potential of Au-Ns. **e.** Particle size distribution of AuNPs. **f.** Particle size distribution of Au-Ns. **g.** TEM image of AuNPs. **f.** TEM image of Au-Ns.

The synthesized AuNPs and Au-Ns showed a typical peak of red-wine gold nanoparticles at 519 nm and 520 nm **(**Fig. 2**a, b)**. The zeta potential for AuNPs and Au-Ns were –22.37 ± 1.02 and −13.42 ± 1.12 mV, respectively **(**Fig. 2**c, d)**. Furthermore, DLS analysis showed size distribution of AuNPs ranged from 8 to 14 nm, with a main peak at 10 nm. Meanwhile, the Au-Ns showed particle size distribution ranging from 11 to 20 nm with a main peak at 17 nm **(**Fig. 2**e, f)**. The TEM images showed spherical shape particles of AuNPs and Au-Ns with a size range from 6 −11 nm and 8–20 nm, respectively **(**Fig. 2**g, h).**

**Fig. 3:**
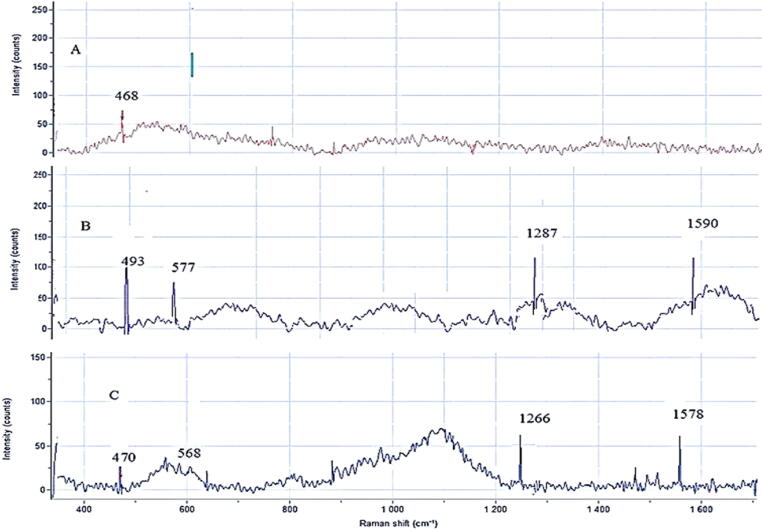
Raman Spectroscopic analysis, **A**. AuNPs (Peak at 468 cm^−1^), **B**. DNA probe (peaks at 493, 577, 1287, and 1590 cm^−1^ corresponding to 2PO-, C—O, and C—C vibrations); **C**. AuNPs-DNA probe (peaks at 481, 568, 1266 and 1578 cm^−1^).

The Raman spectrum of the thiolated oligonucleotide showed characteristic peaks at 493, 577, 1287, and 1590 cm^−1,^ corresponding to 2PO-, C—O, and C—C vibrations, respectively. Similar peaks have appeared in the AuNPs-DNA probe at 481, 568, 1266, and 1578 cm^−1^. AuNPs showed one peak at 468 cm^−1^ **(**Fig. 3**).**

**Fig. 4:**
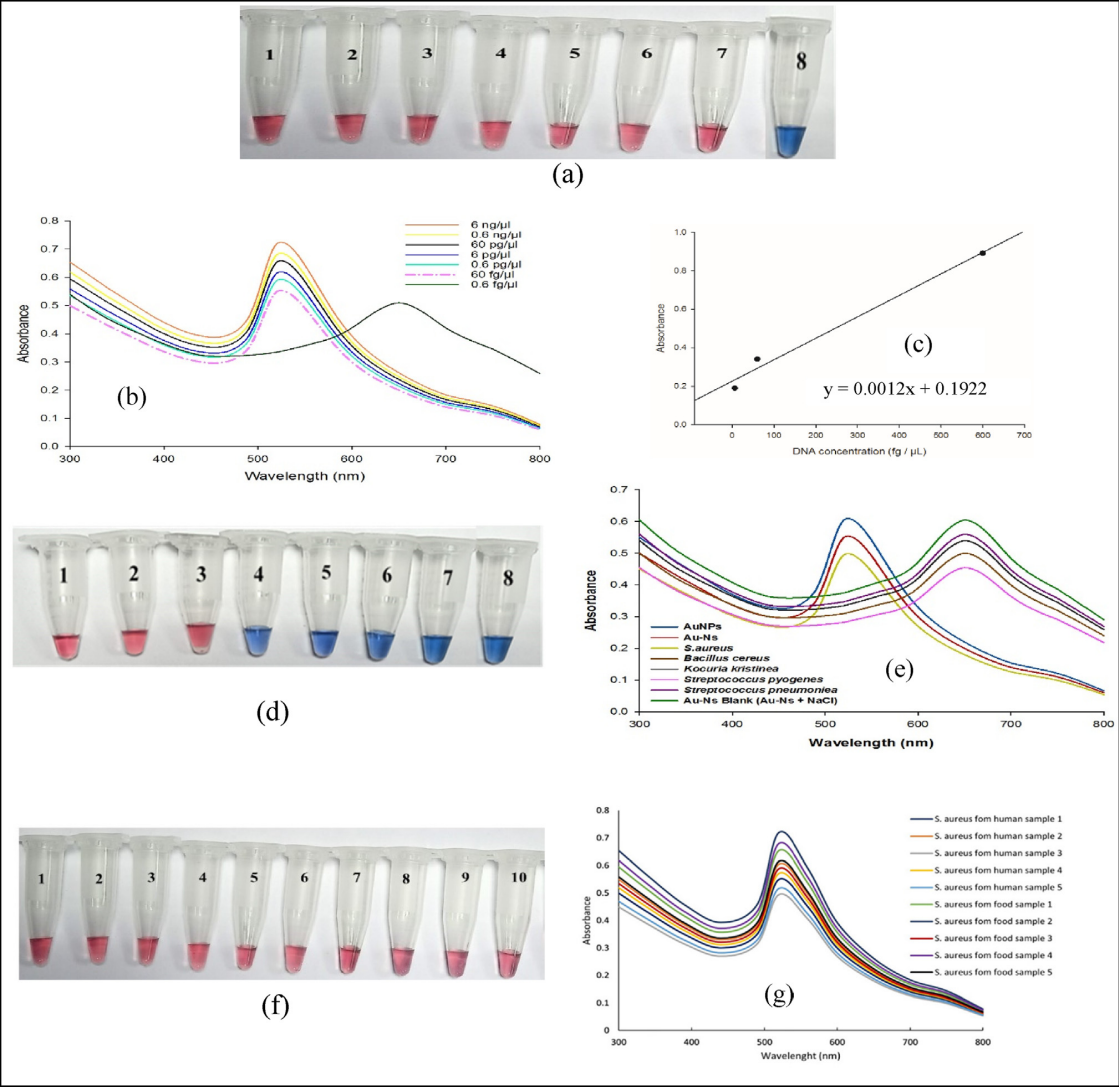
**a, b**. representative image and UV–vis spectra of the colorimetric assay of the *S. aurous* standard samples at different concentrations of the target DNA. From 1 → 8 (6 ng/μl, 0.6 ng/μl, 60 pg/μl, 6 pg/μl, 0.6 pg/μl, 60 fg/μl, and 6 fg/μl). **c.** Linear relationship between the different concentrations of the target DNA and absorbance changes from 6 to 600 fg/μL. **d, e.** representative image and UV–vis spectra of the colorimetric assay for *SPA* gene by Au-Ns in the positive and negative control. (**1**. Au-Ns**; 2**. AuNPs**; 3**. *S. aureus***; 4**. *Bacillus Cereus***; 5**. *Kocuria Kristinae***; 6**. *Streptococcus Pyogenes***; 7**. *Streptococcus Pneumoniae*; **8**. Au-Ns + NaCl). The red color illustrates the detection of the target sequence. **f, g.** representative image and UV–vis spectra of the representative *S. aureus* strains isolated from clinical specimens (1,2,3,4,5) and food samples (6,7,8,9,10).

### 3.3 Colorimetric detection of *SPA* gene

this study was conducted to investigate using AuNPs in the colorimetric detection of the *SPA* gene. The preliminary experiment was performed as reported in the literature **(**Kalidasan et al., 2013, Socohou et al., 2021**)**. However, the current method showed an optimum salt concentration of 1 M and an incubation temperature of 53 °C. Under these conditions, all *S. aureus* standard samples showed a stable red color after adding the salt.

### 3.4 Methods of validation

Under the optimum conditions, The concentration (6 ng/μl, 0.6 ng/μl, 60 pg/μl, 6 pg/μl, 0.6 pg/μl, 60 fg/μl, and 6 fg/μl) of *S. aureus* reference standard samples revealed red-colored solutions that indicate the presence of the *SPA* gene, while the concentration 0.6 fg/μl provide blue color. The developed color was stable for eight hours, and the results were verified by UV–Vis spectrophotometer, as shown in **(**Fig. 4**b).** According to the equation)1(and the calibration curve **(**Fig. 4**c),** the DL of the colorimetric assay equals 6 fg/µL.

Furthermore, positive and negative samples, along with blank, were examined to determine the method specificity **(**Fig. 4**c, d)**. After adding 1 M NaCl, the solution containing *S. aureus* genomic DNA remains red with a nonsignificant red-shift to a larger wavelength of 522 nm. On the other hand, the color of Au-Ns blank and negative control samples turned blue immediately after NaCl addition and exhibited a significant absorbance shift towards a longer wavelength (650 nm).

Finally, the storage stability was determined by regular daily usage of Au-Ns with a freshly extracted DNA sample for thirty days. Results showed excellent stability of the prepared Au-Ns in detecting the *SPA* gene with the same detection limit. At the end of this period, the Au-Ns color spontaneously shifted to purple, so it is not suitable for use after 30 days of storage.

### 3.5 Direct application on clinical and food samples

In the current study, 40 *S. aureus* isolates were obtained from collected samples (20 food samples and 50 clinical specimens) using the Baird Parker agar medium. *S. aureus* isolates were preliminarily identified via microscopical examination, biochemical tests and PCR analysis. Further confirmation was performed using the synthesized Au-Ns for detecting the *S. aureus SPA* target gene. The obtained results revealed stable red color with isolated DNA samples of all tested isolates, Fig. 4**(e, f)** shows five representative *S. aureus* strains isolated from clinical specimens and five isolated strains from food samples.

## 4 Discussion

For epidemiological investigations, prevention, and control of infections, molecular typing is crucial. Choosing the method for molecular typing is based on simplicity, consistency, cost, and easiness of interpretation. Sequence typing of the *SPA* gene repeat region was used to study the epidemiology of methicillin-resistant *Staphylococcus aureus* **(**MRSA) **(**Harmsen et al., 2003, Fasihi et al., 2017**).**

Protein A, found in all strains of *S. aureus,* is the most studied cell surface protein in Gram-positive bacteria encoded by the *SPA* gene, which can be used as a genetic marker to detect *S. aureus* by PCR **(**Kim et al., 2012**)**. Our results showed successful identification of the *SPA* gene with a molecular size previously reported in the literature **(**Houhoula et al. 2017**)**.

The chemical method of AuNPs synthesis exhibits attractive properties, such as simplicity, high yield, low cost, and stability of the obtained nanoparticles (Liu et al., 2014). According to the literature, DTT activation is essential in AuNPs-probe conjugation **(**Narmani et al., 2018, Elahi et al., 2019**).** Herein, the successful synthesis of AuNPs and Au-Ns was confirmed via different physicochemical characterization techniques. As previously mentioned, the Color of AuNPs is sensitive to their size and aggregation **(**Verma et al., 2015**)**, so it is essential to characterize the obtained AuNPs and Au-Ns in the current study. The results revealed good stability and well-dispersed uniform particle size of the obtained nanomaterials indicated by zeta potential, DLS analysis, and TEM imaging.

Raman spectroscopy is a commonly used spectroscopic technique to provide a structural fingerprint of the organic molecules **(**Narmani et al., 2018**)**. Herein Raman spectroscopy was used to confirm the successful conjugation of AuNPs with the thiolated oligonucleotide. Herein, the presence of similar peaks in both AuNPs and Au-nanosensor Raman spectra confirmed the successful conjugation of AuNPs with the thiolated oligonucleotide. Similar results were reported in the literature **(**Verma et al., 2015, Narmani et al., 2018**).**

Colorimetric detection of DNA by using oligonucleotide-functionalized AuNPs was firstly described by Mirkin et al. in 1996. Indeed, AuNPs are easy to synthesize, simple to modify, and highly biocompatible (Yeh et al., 2012). Moreover, AuNPs have a high affinity to the thiol group, so they could be easily functionalized with thiolated oligonucleotides without amplifying this targeted DNA **(**Baptista et al., 2011, Houhoula et al., 2017**)**. Depending on this phenomenon, AuNPs-based sensors have been extensively used for detecting specific DNA sequences. The colorimetric assay of DNA using Au-Ns is performed in two ways **(**Truong et al., 2012**).** Firstly, the cross-linking method in which AuNPs are conjugated to the target nucleic acid strand with two probes. The presence of the target sequence causes particle aggregation by cross-linking AuNPs together and vice versa **(**Verma et al., 2015**).** On the other hand, the non-cross-linking method includes binding AuNPs with a single thiolated probe before hybridization. In this situation, binding the target to the probe results in a double helix formation, causing high stability against aggregation after adding NaCl or HCl **(**Dharanivasan et al., 2016, Shahbazi et al., 2018**)**. Consequently, the absence of the target leads to particle aggregation under the same conditions. In the current study, a single probe was designed for *SPA* gene detection with sequence (5′SH-GCCTAACTTGAACGAAGAAC3′). In contrast, Houhoula et al. (2017) used a probe with sequence 5′SH-CATTAGTGGAAAGATGGAATG3′. However, our designed probe seemed to have a higher affinity to the target gene, as revealed by the stability of Au-Ns after the addition of concentrated NaCl. Moreover, the non-crosslinking assay is affected by four main factors: incubation temperature, size of AuNPs, probe sequence, and salt concentrations. In this study, the incubation temperature and salt concentration were optimized to obtain the optimum conditions for *SPA* gene detection.

The detection limit is the lowest quantity of a substance that can be distinguished from the absence of that substance **(**Elahi et al., 2019**).** Based on optimal assay conditions, the DL was determined with serially diluted DNA samples. Compared with PCR based method (DL 60 pg/μL), using Au-Ns in *SPA* gene detection may be a promising approach to achieve higher sensitivity and early diagnosis of *S. aureus*.

Specificity is the ability of the probe to bind a specific target without interference. The Au-Ns was designed to selectively detect the *S. aureus SPA* gene. So, The color and the absorbance of the AuNPs, Au-Ns, were compared with the tested DNA from *S. aureus*, *Streptococcus Pyogenes, Streptococcus Pneumoniae*, *Kocuria kristinae*, and *Bacillus cereus* by naked eye and UV–vis spectrophotometry as mentioned above, the AuNPs and Au-Ns had a wine red color and maximum absorbance at 519/520 nm. In agreement with all previous reports **(**Houhoula et al., 2017, Shahbazi et al., 2018, Amini et al., 2018**)** the designed probe showed only positive results with the target gene, and no false-negative results were recorded. So, the specificity % of Au-Ns is 100 %, as calculated by Eq. (2).

The presence of *S. aureus* in clinical specimens and food samples represents a potential health risk and is challenging for both diagnosis and therapeutic strategies **(**Pérez et al., 2018, Socohou et al., 2021**)**. Therefore, it is essential to use a quick and accurate technique to detect toxigenic strains of *S. aureus* to evaluate the human health risk arising from healthcare and community-associated infections. *S. aureus* gives characteristic shiny black colonies with white precipitation on the Baird Parker media **(**Corry et al., 2003**)**, so this media is selectively used to primary isolate and identify it.

## 5 Conclusion

In conclusion, the Au-Ns can be utilized as a highly sensitive and specific tool for sensing *S. aureus.* The proposed assay had several advantages in detecting DNA samples. First, the detection was rapid and convenient as it required 20 min for test completion after genomic DNA extraction without DNA amplification. Afterward, the results were recognized visually and UV–vis spectrophotometry before and after salt-induced Au-Ns aggregation. Second, Au-Ns synthesis is cost-effective and straightforward, so the need for complicated experimental techniques and equipment could be omitted. Third, the detection limit was lower than previously reported conventional methods or AuNPs based sensors. Also, the proposed assay was applied on reference standard strain, pure culture isolates, clinical and food samples, in a better performance than PCR amplification, without using complex instruments and infrastructure. Finally, the suggested method represents a generic AuNPs based platform technology with the potential point of care application for clinical detection of many diseases or food pathogens. As a recommendation, this method has to conduct a clinical trial with a greater sample size to evaluate clinical sensitivity and specificity in both the developed and developing worlds.

## Declaration of Competing Interest

The authors declare that they have no known competing financial interests or personal relationships that could have appeared to influence the work reported in this paper.
